# Epitope-Based Vaccines: The Next Generation of Promising Vaccines Against Bacterial Infection

**DOI:** 10.3390/vaccines13030248

**Published:** 2025-02-27

**Authors:** Jing Li, Yan Ju, Min Jiang, Sha Li, Xiao-Yan Yang

**Affiliations:** School of Bioengineering, Zhuhai Campus of Zunyi Medical University, Zhuhai 519041, China; lijing@zmuzh.edu.cn (J.L.);

**Keywords:** epitope-based vaccines, B-cell epitopes, T-cell epitopes, bacterial vaccines, *Staphylococcus aureus*

## Abstract

The increasing resistance of bacteria to antibiotics has underscored the need for new drugs or vaccines to prevent bacterial infections. Reducing multidrug resistance is a key objective of the WHO’s One Health initiative. Epitopes, the key parts of antigen molecules that determine their specificity, directly stimulate the body to produce specific humoral and/or cellular immune responses. Epitope-based vaccines, which combine dominant epitopes in a rational manner, induce a more efficient and specific immune response than the original antigen. While these vaccines face significant challenges, such as epitope escape or low immunogenicity, they offer advantages including minimal adverse reactions, improved efficacy, and optimized protection. As a result, epitope-based vaccines are considered a promising next-generation approach to combating bacterial infections. This review summarizes the latest advancements, challenges, and future prospects of epitope-based vaccines targeting bacteria, with a focus on their development workflow and application in antibiotic-resistant pathogens with high mortality rates, including *Staphylococcus aureus*, *Streptococcus pneumoniae*, *Streptococcus pyogenes*, *Klebsiella pneumoniae*, *Acinetobacter baumannii*, and *Pseudomonas aeruginosa*. The goal of this review is to provide insights into next-generation vaccination strategies to combat bacterial infections associated with antibiotic resistance and high mortality rates.

## 1. Introduction

The growing resistance and persistence of bacteria to antibiotics pose a significant global threat to human health, resulting in substantial burdens of morbidity and mortality [1,2,3]. This issue is in line with the World Health Organization’s (WHO’s) One Health initiative, which emphasizes the importance of reducing multidrug-resistant infections through coordinated global efforts. The six leading bacteria associated with resistance-related deaths include *Escherichia coli*, *Staphylococcus aureus*, *Klebsiella pneumoniae*, *Streptococcus pneumoniae*, *Acinetobacter baumannii*, and *Pseudomonas aeruginosa* [1]. For instance, methicillin-resistant *S. aureus* (MRSA) alone accounts for over 100,000 deaths annually worldwide, highlighting the urgent need for innovative solutions to combat antibiotic-resistant infections [4].Vaccination is considered one of the most effective strategies to prevent and control bacterial infections, especially in the context of antibiotic resistance. Although traditional live attenuated vaccines and inactivated vaccines induce durable immune responses, their production involves extensive processes, including the removal of harmful chemicals or the dilution of inactivated forms, which can be cumbersome and time-consuming. Moreover, the modification of antigens during inactivation may adversely affect vaccine efficacy, necessitating the production of larger quantities of antigens to elicit adequate immune responses, with associated safety risks. This not only drives up production costs but also raises safety concerns, particularly for vulnerable populations, such as the elderly and immunocompromised individuals [5,6].

In contrast, DNA, mRNA, and protein vaccines are easier to manufacture and present improved safety profiles. Epitopes, also referred to as antigenic determinants, are crucial for antigen specificity and are recognized by the corresponding paratope of antibodies, triggering specific humoral and/or cellular immune responses. A well-designed combination of dominant epitopes can elicit a more effective immune response than the original antigen. Identifying antigens or epitopes that enhance immune response stimulation remains a major challenge in the development of effective protein, DNA, and mRNA vaccines [7]. Fortunately, advances in reverse vaccinology and bioinformatics have enabled time- and cost-effective vaccine design and development [8]. Consequently, epitope-based vaccines have emerged as promising strategies, garnering increasing attention in the field.

Epitope-based vaccines can be formulated as DNA, mRNA, or protein vaccines, which offer distinct advantages, including minimal adverse reactions, enhanced efficacy, and optimized protection, particularly for vulnerable populations such as the elderly and immunocompromised individuals, when compared to traditional vaccines (Table 1) [7,8,9,10]. Given the limited serotype coverage and risk of epitope escape associated with single-epitope peptides, employing multiple epitopes that target single or multiple proteins is considered an ideal approach to designing epitope-based vaccines [7].

The first epitope-based vaccine targeted cholera toxin and the heat-labile toxin of *Vibrio cholerae* and *E. coli*. Immunization with a fusion protein containing a cholera toxin short peptide (CTP3, regions 50–64 of the B subunit of cholera toxin) and β-galactosidase can elicit neutralizing antibodies against both the cholera toxin and the heat-labile toxin of *E. coli*, demonstrating the potential of epitope-based strategies [11]. Recently, significant focus has been placed on developing epitope-based vaccines to combat bacterial infections associated with antibiotic resistance and high mortality rates. The aim of this review is to comprehensively explore the design processes and applications of epitope-based vaccines targeting six critical bacterial pathogens linked to antibiotic resistance and high mortality rates, including *S. aureus*, *S. pneumoniae*, *S. pyogenes*, *K. pneumoniae*, *A. baumannii*, and *P. aeruginosa*. Furthermore, we outline current challenges and future directions for researchers engaged in optimizing epitope-based vaccines against these formidable bacterial infections.

## 2. Design and Validation Processes of Epitope-Based Vaccines

**Figure 1:**
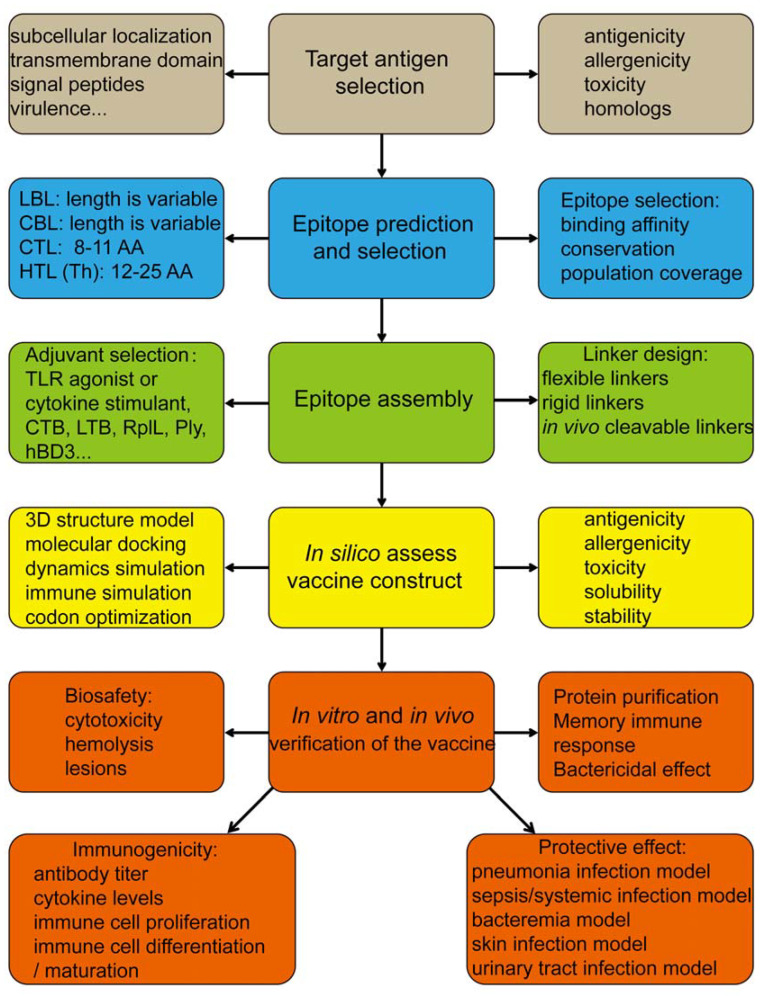
The procedure for developing bacterial epitope-based vaccines. LBL, linear B lymphocyte epitope; CBL, conformational B lymphocyte epitope; CTL, cytotoxic T lymphocyte epitope; HTL, helper T lymphocyte epitope; TLR, Toll-like receptor; CTB, cholera toxin subunit B; LTB, heat-labile enterotoxin B; RplL, 50S ribosomal protein L7/L12; Ply, pneumolysin; hBD3, human beta-defensin 3.

As an interdisciplinary field, bioinformatics provides crucial tools for the *in silico* prediction and analysis of epitope-based vaccines. The rational workflow for epitope-based vaccine design is illustrated in Figure 1, which includes target antigen selection, epitope prediction and selection, epitope assembly, an assessment of the fusion epitope-based vaccine construct *in silico*, and the verification of the biosafety and efficiency of the epitope-based vaccine *in vitro* and *in vivo*.

### 2.1. Target Antigen Selection

Bacterial organisms contain thousands of proteins; however, only a limited number of conserved proteins can serve as effective vaccine antigens capable of eliciting a protective immune response. Therefore, the accurate selection of target antigens is a critical first step in designing epitope-based vaccines. Various bioinformatics tools, including decision trees, filtering algorithms, and machine learning (ML) approaches, are employed to predict potential target antigens from bacterial genomes or proteomes [12,13,14]. Among these, machine learning tools such as VaxiJen and Bowman–Heinson typically outperform decision tree and filtering algorithms.

VaxiJen is the first server utilizing ML algorithms for the alignment-independent prediction of protective antigens. It identifies relatively conserved antigens across bacteria, viruses, tumors, parasites, and fungi. The dataset used by VaxiJen to predict bacterial immunogens consists of 317 experimentally validated immunogenic proteins collected from research publications and the corresponding protein sequences obtained from NCBI and UniProtKB [15]. The antigenicity score generated by VaxiJen considers multiple factors, including the physical and chemical properties of proteins, structural features, and interactions with major histocompatibility complex (MHC) molecules. A protein is classified as a protective antigen if its antigenicity score exceeds a threshold of 0.4 [14].

Furthermore, Vaxign2, a second-generation network vaccine design program based on reverse vaccinology and ML, complements the results obtained from VaxiJen [16]. Proteins that are cell wall-associated, extracellular, secreted, membrane-associated, and nonallergenic or nontoxic are more likely to be recognized as protective antigens compared to cytoplasmic or intracellular proteins. This is because surface-exposed or secreted proteins are more accessible to immune recognition and often contain epitopes critical for pathogen neutralization. Therefore, factors such as subcellular localization, transmembrane domains, signal peptides, virulence, allergenicity, and toxicity are predicted to identify optimal vaccine candidates [7,17]. Additionally, the homologs of protective antigens within the vaccine recipient host are assessed using BLASTp (https://blast.ncbi.nlm.nih.gov/Blast.cgi (accessed on 27 January 2025)) to mitigate the risk of autoimmunity [17].

### 2.2. Epitope Prediction and Selection

Epitopes are pivotal in determining the specificity of target antigens that trigger specific humoral and/or cellular immune responses. The proper prediction and selection of epitopes constitute the most critical components of epitope vaccine development. Epitopes can be classified as B-cell epitopes (BCEs) and T-cell epitopes (TCEs). BCEs are recognized by B-cell receptors (BCRs) and can also be presented by MHC class II molecules, where the complexes bound to MHC II are recognized by BCRs on the cell surface. Conversely, TCEs are recognized by MHC I or MHC II within the cell, presented on the cell surface, and subsequently recognized by the T-cell receptors (TCRs) of CD8+ cytotoxic T cells and CD4+ helper T cells, respectively [18,19].

The bioinformatics prediction of BCEs and TCEs primarily involves predicting the binding affinity of epitope peptides to MHC I or MHC II by using data-driven approaches [20,21]. BCEs can be divided into linear B lymphocyte epitopes (LBLs, also referred to as continuous epitopes, constituting 10% of BCEs) and conformational B lymphocyte epitopes (CBLs, or discontinuous epitopes, accounting for 90% of BCEs) [22]. Linear epitopes consist of contiguous amino acids on the peptide chain, whereas conformational epitopes comprise amino acids that are spatially proximate but discontinuous. The identification of conformational epitopes is often more challenging given the difficulty in resolving many bacterial proteins using X-ray crystallography or NMR, and the native structure may not be easily reproduced without a protein scaffold. Consequently, current epitope vaccine designs focus predominantly on linear epitopes. Research has indicated that the size of an antibody pair that typically binds to BCEs ranges from 15 to 22 structural amino acids [23,24]. Tools such as BepiPred (https://services.healthtech.dtu.dk/service.php?BepiPred-3.0 (accessed on 27 January 2025)), ABCpred (https://webs.iiitd.edu.in/raghava/abcpred/ (accessed on 27 January 2025)) [25], and IEDB B-cell epitopes (http://tools.iedb.org (accessed on 27 January 2025)) [26] are available for LBL prediction, whereas ElliPro (http://tools.iedb.org/ellipro/ (accessed on 27 January 2025)) [27] and DiscoTope (https://services.healthtech.dtu.dk/service.php?DiscoTope-3.0 (accessed on 27 January 2025)) [28] are utilized for CBL prediction.

T-cell epitopes can be divided into cytotoxic T lymphocyte epitopes (CTLs) recognized by MHC I and the TCRs of CD8+ cytotoxic T cells and helper T lymphocyte epitopes (HTLs or Ths) recognized by MHC II and the TCR of helper CD4+ T cells. Unlike the variable length of BCEs, TCEs are typically fixed-length short linear peptides: those binding to MHC I are typically 8–11 AA long, whereas those binding to MHC II are typically 12–25 AA long [29,30]. There are two methods used for predicting TCEs: direct and indirect. The indirect method is primarily used to predict TCEs using peptide binding to MHC molecules, with CTLPred being the only known program that uses the direct method to predict CTLs based on the recognition of short peptides by T cells [17,31]. Several methods and tools have been established to forecast TCEs, including IEDB MHC-I Binding (http://tools.immuneepitope.org/mhci/ (accessed on 27 January 2025)), IEDB MHC-II Binding (http://tools.immuneepitope.org/mhcii/) [32], NetMHCpan (https://services.healthtech.dtu.dk/services/NetMHCpan-4.1/ (accessed on 27 January 2025)) [33], and NetMHCIIpan (https://services.healthtech.dtu.dk/services/NetMHCIIpan-4.0/ (accessed on 27 January 2025)) [34].

In addition to binding affinity, the development of an efficient epitope-based vaccine that can protect a broader target population should involve selecting epitopes relatively conserved among different bacterial serotypes that bind to multiple MHC alleles of the target host. Several programs are available for epitope peptide conservancy and population coverage, including IEDB Population coverage, IEDB Epitope conservancy, IEDB-clustering analysis (https://www.iedb.org/ (accessed on 27 January 2025)), and BLAST (https://blast.ncbi.nlm.nih.gov/Blast.cgi (accessed on 27 January 2025)) [17].

### 2.3. Epitope Assembly

The selected epitopes are assembled into a vaccine construct, which may include adjuvant selection and linker design. To augment epitope-based vaccine induced immune responses and enhance protection against bacteria, adjuvants are essential. Adjuvants often act as Toll-like receptor (TLR) agonists or cytokine stimulants, boosting the magnitude, breadth, and durability of the immune response when placed at the N-terminus or C-terminus *via* the EAAAK linker within a vaccine construct [35]. Common adjuvants used for epitope-based vaccine development include cholera toxin subunit B (CTB) [36], heat-labile enterotoxin B (LTB) [37], 50S ribosomal protein L7/L12 (RplL) [38], pneumolysin (Ply) [39], and human beta-defensin 3 (hBD3) [40].

Besides epitopes and adjuvants, linkers are indispensable components in the construction of epitope-based vaccines. The selection of suitable linkers can improve structural stability, facilitate antigen presentation, prevent the formation of additional nonscreened epitopes, improve biological activity, increase expression yield, and achieve desirable pharmacokinetic profiles [9,41]. Linkers are categorized into three categories: flexible linkers, rigid linkers, and *in vivo* cleavable linkers. Flexible linkers usually enable effective separation between selected epitopes, including sequences such as (GGGGS)n, (G)n, KK, AAY, GPGPG, EGKSSGSGSESKST, and GSAGSAAGSGEF. Rigid linkers maintain epitope functionality and structural stability through constrained spacing, including sequences such as (EAAAK)n and (XP)n (X: alanine, lysine, or glutamine) [9,41,42]. *In vivo* cleavable linkers not only target specific sites within the host but also enable multi-epitope vaccines to be processed and cleaved into individual epitopes, employing disulfide and protease-sensitive sequences [41]. The SynLinker web server is designed to create linkers for fusion proteins based on the linker length, AA composition, solvent accessibility, the absence of protease sensitive sequences, and other user-specific parameters [43].

### 2.4. Assessment of Fusion Epitope-Based Vaccine Construct In Silico

The final fusion epitope-based vaccine construct, composed of epitope peptides, linkers, and/or adjuvants, is folded into a tertiary structure and presented to the immune system. Similar to protein antigens, its antigenicity, allergenicity, and toxicity must be evaluated. In addition to exhibiting high antigenicity and being nonallergenic and nontoxic, an optimal epitope-based vaccine construct should possess stability, hydrophilicity, good water solubility, and high thermostability [9,17]. These physicochemical properties can be assessed using the ProtParam web server (https://web.expasy.org/protparam/ (accessed on 27 January 2025)).

The 3D structure model of the fusion epitope-based vaccine construct is predicted and validated through bioinformatics, including molecular docking, molecular dynamics simulation, and immune simulation [7,9,17]. Homology modeling is the most reliable approach for predicting the 3D structure of epitope-based vaccine proteins. Key tools for homology modeling include the following:

- AlphaFold [44]: Achieves near-experimental accuracy by integrating deep learning with evolutionary-scale multiple sequence alignments. Ideal for *de novo* prediction when templates are scarce.
- SWISS-MODEL [45]: A template-based automated server using PDB templates, ideal for the rapid modeling of proteins with high sequence similarity (>30%).
- I-TASSER [46]: Combines threading and *ab initio* simulations, suitable for novel epitope scaffolds (TM-score > 0.5 indicates correct topology).
- Raptor-X [47]: Employs deep learning to predict contact maps, achieving high accuracy (F1-score > 0.8) for membrane-bound antigens.

To trigger both innate and adaptive immune responses against infectious bacteria, antigens should interact with host immune receptors, such as Toll-like receptors (TLR2 and TLR4) [48]. The interactions between the best 3D predicted models of the epitope-based vaccine construct and TLR2 or TLR4 are determined using molecular docking and molecular dynamics simulation. Molecular docking detects the best-docked intermolecular conformations and binding free energy between the vaccine construct and the TLR2 or TLR4 receptors. Commonly used docking tools include the following:

- PatchDock (https://bioinfo3d.cs.tau.ac.il/PatchDock/ (accessed on 27 January 2025)) [49]: A geometry-based algorithm for protein–protein docking (speed: ~1000 complexes/h).
- AutoDock Vina (https://github.com/ccsb-scripps/AutoDock-Vina (accessed on 27 January 2025)) [50]: A flexible ligand docking tool with high-throughput capabilities (binding affinity prediction error < 2.8 kcal/mol).
- HDOCK (http://hdock.phys.hust.edu.cn/ (accessed on 27 January 2025)) [51]: A hybrid server integrating template and *ab initio* approaches (ranked first in CAPRI blind tests).

Molecular dynamics simulation further assesses docking binding stability, binding free energy, and residual flexibility, using metrics such as the root mean square derivative (RMSD) and root mean square fluctuation (RMSF) [17]. Common tools for these simulations include the following:

- GROMACS (https://www.gromacs.org (accessed on 27 January 2025)) [52]: An open-source, GPU-accelerated software designed for large systems (>1 million atoms).
- AMBER (https://ambermd.org/ (accessed on 27 January 2025)) [9,17]: A force field with validated accuracy for nucleic acids and membrane proteins.

Constructs with negative binding free energies and lower RMSD and RMSF values are considered more stable. Additionally, immune simulation assesses whether the constructed vaccine can induce a robust immune response. Two common tools for immune simulation are the following:

- C-ImmSim [53,54]: An open-source server for predicting immunoglobulin levels (including subclasses), Th cell development, and cytokine profiles.
- IEDB Class I Immunogenicity [55]: Provides a score indicating the immunogenicity of a peptide when presented on an MHC class I molecule.

Beyond immunogenicity, immune simulation can optimize the dosage, formulation, and scheduling of epitope-based vaccine constructs.

Finally, to ensure efficient protein expression, the codons in the vaccine construct should be optimized for the specific host organism (e.g., *E. coli*, mammalian cells, yeast, or insect cells) used for foreign gene expression [17,56]. Codon optimization can be performed using tools such as the following:

- Java Codon Adaptation Tool (JCat) (https://www.jcat.de (accessed on 27 January 2025)) [57]: Optimizes the codon adaptation index (CAI > 0.8) and removes cryptic splice sites.
- GenSmart Codon Optimization (https://www.genscript.com/gensmart-free-gene-codon-optimization.html (accessed on 27 January 2025)) [9]: Customizes GC content (30–70%) and avoids restriction enzyme sites.

### 2.5. Verification of Biosafety and Efficacy of Epitope-Based Vaccines In Vitro and In Vivo

After codon optimization, the DNA of the final optimized vaccine construct should be inserted into an appropriate expression vector. The fusion protein of the vaccine construct is then induced and expressed in a suitable host organism known for high expression efficiency and ease of purification. It is essential to incorporate specific tags, such as His and GST tags, to facilitate effective purification. The resulting fusion protein is purified and identified using affinity chromatography techniques (e.g., Ni-NTA and GST affinity chromatography) and sodium dodecyl sulfate polyacrylamide gel electrophoresis (SDS-PAGE). Following purification, the fusion protein, mixed with or without an adjuvant such as aluminum hydroxide, is inoculated into mice, rabbits, or other animals *via* intranasal, subcutaneous, intramuscular, or oral administration. A set of common methods is then used to validate the biosafety and efficacy of epitope-based vaccines, including assessments of immunogenicity, memory immune, protective effects, and bactericidal effects [58].

The biosafety of the vaccine construct was evaluated to determine whether it has any detrimental effects on the intended vaccine recipients. The key metrics for biosafety evaluation include the following: (1) cytotoxicity—an assessment of the impact of the vaccine construct on host cells; (2) hemolysis—an evaluation of the effects of the vaccine construct on host red blood cells; and (3) lesions—an assessment of damage to host tissues such as the heart, liver, spleen, lungs, and kidneys.

Immunogenicity is a critical characteristic of a vaccine, reflecting its ability to trigger cellular and/or humoral immune responses in recipients. Indicators for assessing the immunogenicity of bacterial epitope-based vaccines include the following: (1) antibody titer—measuring specific antibody titers, including IgG, IgA, IgE, and IgG subclasses (e.g., IgG1, IgG2), using an enzyme-linked immunosorbent assay (ELISA); (2) cytokine levels—measuring cytokines (e.g., IFN-γ, IL-2, IL-4, IL-6, IL-10, and IL-17) *via* an ELISA, enzyme-linked immunospot assay (ELISPOT), or flow cytometry; (3) immune cell proliferation—using fluorescent dyes (e.g., CFSE) to track cell division through flow cytometry, alongside counting cells before and after stimulation or employing indirect methods based on the tetrazolium/formazan reaction in MTT or CCK8 assays [54,59]; and (4) immune cell differentiation/maturation—assessing specific markers of the differentiation and maturation of immune cells, such as B cells, T cells, and dendritic cells (DCs), through flow cytometry. These metrics are also used for optimizing the dosage, formulation, and schedule of the epitope-based vaccine construct.

A desirable characteristic of epitope-based vaccines is the establishment of a memory immune response. The efficacy of a vaccine relies on the effective immune memory generated post-vaccination. Upon re-exposure to the same antigen or pathogen, memory cells, including memory B cells and CD4+ and CD8+ memory T cells, mount an antigen-specific immune response, thereby facilitating effective immune protection [60,61]. Memory B cells and CD4+ and CD8+ memory T cells can be analyzed using flow cytometry, ELISA, or ELISPOT.

The protective effect is another key aspect of bacterial epitope-based vaccines. Common animal challenge models for evaluating the protective effects of vaccine constructs against pathogens such as *S. aureus*, *S. pneumoniae*, *S. pyogenes*, *K. pneumoniae*, *A. baumannii*, and *P. aeruginosa* include the following: (1) pneumonia infection model—challenge with bacteria *via* intranasal (i.n.) administration [62,63]; (2) sepsis/systemic infection model—challenge with bacteria *via* intraperitoneal (i.p.) administration [54,59,63]; (3) bacteremia model—challenge with bacteria *via* tail intravenous (i.v.) administration [64]; (4) skin infection model—challenge with bacteria *via* subcutaneous administration [65,66]; and (5) urinary tract infection (UTI) model—challenge with bacteria *via* transurethral bladder administration [67]. In addition to survival rates, bacterial loads in blood or major tissues and the pathological status of these tissues are also assessed. Additionally, the bactericidal effects of antibodies or serum are evaluated using an opsonophagocytic killing activity (OPK) assay [68]. Epitope-based vaccine candidates showing good immunogenicity and protective effects in animal models will be tested in clinical trials to determine their safety, immunogenicity, and protective efficacy in humans [69].

## 3. Advances in Paradigms for the Development of Epitope-Based Vaccines Against Bacteria

**Figure 2:**
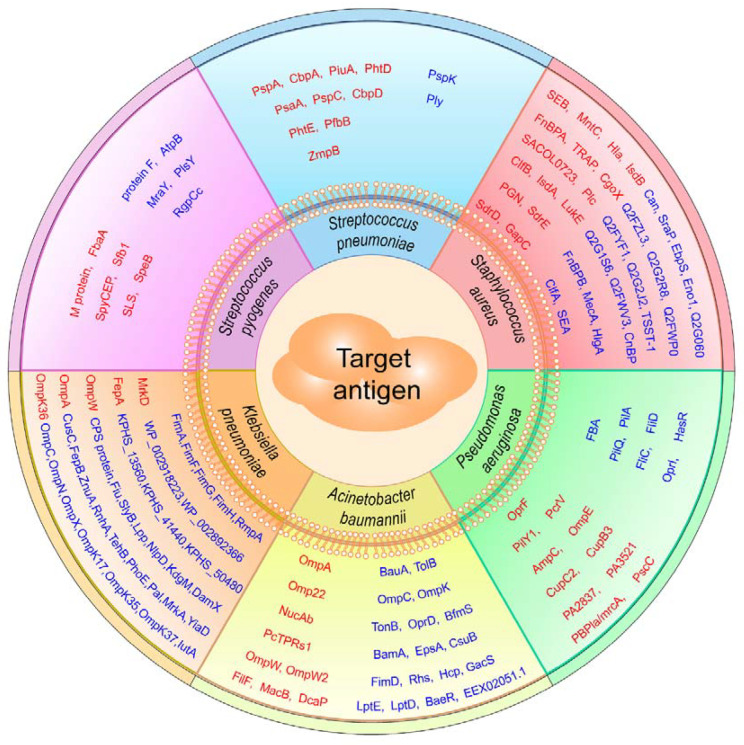
Summary of known target antigens (highlighted in red) used for designing epitope-based vaccines against *S. aureus*, *S. pneumoniae*, *S. pyogenes*, *K. pneumoniae*, *A. baumannii*, and *P. aeruginosa*. Target antigens in red indicate those with known immunogenicity and/or protective effects in animal models. Target antigens in blue indicate those predicted using bioinformatics tools but lacking *in vitro* and *in vivo* experimental validation.

Recently, numerous epitope-based vaccines have been explored to combat various bacterial infections using the strategies mentioned above, and the number of epitope-based vaccines targeting bacteria has rapidly increased. Here, we summarize successful paradigms for the development of epitope-based vaccines against *S. aureus*, *S. pneumoniae*, *S. pyogenes*, *K. pneumoniae*, *A. baumannii*, and *P. aeruginosa* (Table 2 and Figure 2).

### 3.1. Advances in the Development of Epitope-Based Vaccines Against S. aureus

*S. aureus* is a common human pathogen that causes a wide range of infections such as pneumonia, septicemia, bacteremia, skin and soft tissue infections, endocarditis, osteomyelitis, septic arthritis, and toxic shock syndrome [95,96]. The rise of antibiotic-resistant strains, particularly methicillin-resistant *S. aureus* (MRSA), has resulted in substantial mortality and increased treatment costs in hospitalized patients. Therefore, there is an urgent need for a vaccine that can effectively protect against *S. aureus* infections. However, many vaccine candidates, ranging from single-antigen to multiantigen formulations, have faced challenges, including adverse reactions and ineffectiveness in late-stage clinical trials [97,98]. For instance, vaccination with the V710 vaccine (iron surface determinant B, ISdB) was associated with a notable increase in multiple organ failure incidence and mortality among patients undergoing cardiothoracic surgery who developed *S. aureus* infections [99]. Similarly, a four-antigen vaccine (SA4Ag) failed to prevent surgery-associated invasive *S. aureus* infections. The failure of V710 and SA4Ag may be attributed to several factors, including the limitations of animal models in predicting human immune responses, insufficient antigen selection and immune coverage, or potential immunosuppressive reactions interactions different antigens [100]. These findings underscore the challenges in developing an effective vaccine to prevent *S. aureus* infections.

Currently, vaccine development strategies against *S. aureus* have evolved from using entire organisms as immunogens or protein antigens to focusing on epitopes. Various research groups have made significant progress in developing epitope-based vaccines against *S. aureus*. Zou’s lab reported several epitope-based vaccines based on the B-cell epitopes of Staphylococcal enterotoxin B (SEB) [70,101], manganese binding surface lipoprotein C (MntC) [72], alpha-toxin (Hla), and IsdB [73]. As expected, all the SEB-specific multiple B-cell epitope vaccines (polypeptides), MntC-specific single B-cell epitope vaccines (polypeptide mixture of MntC_113–136_-KLH, MntC_209–232_-KLH, and MntC_263–286_-KLH), and Hla/IsdB-specific multiple B-cell epitope vaccines (HI) induced robust IgG titers and evoked protective immune responses against *S. aureus* infection in mouse bacteremia models [70,72,73]. Moreover, the antisera of SEB-specific multiple B-cell epitope vaccines and MntC-specific single B-cell epitope vaccines mediated the opsonophagocytic killing of *S. aureus* in the presence of complement [70,72]. Recently, seven B-cell epitopes (Hla_48–65_, IsdB_402–419_, IsdB_432–449_, SEB_150–167_, SEB_222–239_, MntC_7–24_, SEB_78–95_) from four antigens (Hla, IsdB, SEB, MntC) were identified in a phase 2 clinical trial evaluation of a five-antigen *S. aureus* vaccine (rFSAV). Injection with single monoclonal antibodies (mAbs) against these epitopes provided partial protection in a mouse bacteremia model, while immunization with multiple immunodominant epitope-specific mAbs provided more robust protection against MRSA [71].

Cui’s team identified CD4+ T cell-specific epitopes of MntC. Immunization with one of these epitopes (M8) evoked higher cell proliferation rates and production of IFN-γ, IL-17A, and IL-4, indicating an immune response associated with Th1 and Th17 polarization [102]. However, the M8 epitope did not provide protective effects in a mouse sepsis model [102]. Two linear B-cell epitopes ^272^GYTEDEIVSSD^282^ and ^236^PVATGSLTE^243^ from glyceraldehyde-3-phosphate dehydrogenase C (GapC) were also identified by Cui’s team, and they were shown to provoke a protective humoral immune response against *S. aureus* infection in a mouse sepsis infection model, and their antiserum conferred opsonophagocytic killing activity against *S. aureus* [74,75]. Another linear B-cell epitope (^159^IETFNKANNRFSH^171^) mapped on the N2N3 subdomain of fibronectin-binding protein A (FnBPA) also induced partial protection against *S. aureus* in a mouse sepsis infection model [76]. In 2021, Cui’s team reported an epitope-based vaccine (ATT) based on the Als3-Th-cell epitope of RNAIII Activating Protein (TRAP), which evoked higher levels of IgG, IFN-γ, IL-4, IL-10, and IL-17A and generated a protection effect in immunized mice challenged with lethal doses of *S. aureus* [77].

Additionally, Wang et al. developed a multiple-epitope-based vaccine (MAP27) comprising four epitopes of peptidoglycan (PGN) that elicited T-cell-mediated responses, including the production of IFN-γ, IL-17A/F, and CCL3; reduced bacterial burdens in the organs of BALB/c mice; and generated protective immune responses against *S. aureus* infections in a mouse bacteremia model [78]. Klimka et al. utilized mAb to identify a linear B-cell epitope CgoX-D3 (^377^TDNELVSIVRRD^388^) of coproporphyrinogen III oxidase (CgoX); immunization with the CgoX-D3 epitope conjugated to BSA conferred a 100% protective effect in a murine sepsis model [80]. It is worth noting that a multi-epitope vaccine B, which incorporates the B-cell epitopes from eight well-characterized *S. aureus* virulence factors (ClfB, FnBPA, Hla, IsdA, IsdB, LukE, SdrD, and SdrE), was designed, expressed, and purified. However, immunization with this vaccine failed to induce antibodies capable of recognizing the target antigens and did not confer protection against *S. aureus* infection in mice [58].

Furthermore, multiple-epitope-based vaccines combining B- and T-cell epitopes have been reported. One such vaccine (Hla-MntC-SACOL0723) induced high titers of specific antibodies and elicited Th1, Th2, and Th17 immune responses; however, its ability to generate a protective immune response against *S. aureus* remains untested [103]. In 2020, various epitope-based vaccines containing B- and/or T-cell epitopes of phosphatidylinositol phosphodiesterase (Plc) were constructed, eliciting strong IgG responses and protective immunity in mice challenged with *S. aureus*; notably, the protective effects of the B- and T-cell epitope mixture were significantly superior to those of either the B- or T-cell epitope mixtures alone [79]. Despite promising preclinical results, many of these multi-epitope vaccines require further optimization and extensive clinical trials to confirm their efficacy and safety in humans.

Additionally, many epitope-based vaccines were designed, which comprised the B- and/or T-cell epitopes mapped on FnBPA, collagen adhesin (Cna), serine-rich adhesin for platelets (SraP), and elastin-binding protein (EbpS) [104]; alpha-enolase (Eno1), clumping factor A (ClfA), and IsdB [105]; MntC, IsdA, IsdB, and ClfA [106]; Q2G060, Q2FYF1, and Q2G2J2 [107]; toxic shock syndrome toxin-1 (TSST-1) [108]; Q2FZL3, Q2G2R8, Q2FWP0, Q2G1S6, and Q2FWV3 [109]; ClfA and ClfB [110]; SEA, SEB, and TSST-1 [111]; collagen-binding protein (CnBP) [112]; FnBPA and FnBPB [113]; and transpeptidase (MecA), gamma-hemolysin component A (HlgA), and IsdB [114]. Nevertheless, the immunogenicity and protective effects of the epitope-based vaccines mentioned above still need to be subject to *in vivo* validation or similar.

### 3.2. Advances in the Development of Epitope-Based Vaccines Against S. pneumoniae

*S. pneumoniae* is a significant human pathogen consisting of at least 92 different serotypes, responsible for diseases such as pneumonia, sepsis, and meningitis, especially in children younger than 5 years, the elderly, and immunocompromised individuals [115,116]. Currently, two types of pneumococcal vaccines are available on the market: polysaccharide vaccines (PPVs) and protein-conjugated polysaccharide vaccines (PCVs). However, both PPVs and PCVs are serotype-dependent with limited serotype coverage. Additionally, PPVs, including the 23-valent pneumococcal polysaccharide vaccine (PPV23), do not elicit efficient protective immunity in children younger than 2 years. Meanwhile, PCVs, including PCV7, PCV10, and PCV13, are expensive and complicated to manufacture [117,118,119]. To overcome these drawbacks, epitope-based vaccines based on varied mixtures of highly conserved pneumococcal surface proteins and virulence proteins are introduced as viable alternatives to the present capsular antigen vaccines.

Since pneumococcal surface protein A (PspA) is a crucial virulence factor and widely expressed in all capsular serotypes of *S. pneumoniae*, several reports on the epitope-based vaccines of PspA have demonstrated good immunogenicity and protective effects against *S. pneumoniae* [64,68,81,120]. Four B-cell epitopes based on the proline-rich region of PspA were displayed on the surface of synthetic virus-like particles (SVLPs), which elicited high IgG titers and effective protection against *S. pneumoniae* infection in a mouse bacteremia model [64]. An epitope-based serotype-independent vaccine (PspA1-5c+p), composed of the highest immunodominant coverage of B- and T-cell epitopes derived from the N-terminal sequence of all five PspA clades, induced strong IgG titers and cross-reactivity responses against all pneumococcal serotypes [68]. This vaccine also enabled the antibody-mediated killing of pneumococci *via* the complement system and phagocytic cells. Due to the variability in the N-terminal region of PspA and the potential for serotype-specific epitope escape through mutations [121], multiple-epitope vaccines targeting single or multiple conserved proteins across all *S. pneumoniae* serotypes have been developed.

Dorosti’s team constructed a multi-epitope vaccine comprising CTL epitopes selected from PspA and choline-binding protein A (CbpA) and HTL epitopes from the lipoprotein of the iron uptake ABC transporter (PiuA) and pneumococcal histidine triad protein D (PhtD) [120]. This vaccine elicited high IgG antibody titers and significantly increased the levels of IFN-γ, IL-2, TNF-α, IL-4, and IL-6 while decreasing IL-10 production in mouse sera. Another multi-epitope vaccine based on the B- and Th-cell epitopes of the PspA and PhtD proteins also induced high IgG antibody titers and increased the levels of IFN-γ, IL-4, and IL-17 in mouse sera [81]. This vaccine reduced bacterial loads in blood and spleen tissue and provided good protection in mice after being intraperitoneally challenged with *S. pneumoniae*, and the induced antibodies demonstrated complement-mediated bactericidal activity.

In 2014, six Th-cell epitope peptides (7, 19, 20, 22, 23, and 24) mapped on pneumococcal surface adhesin A (PsaA) were found to induce T-helper cytokine responses (IFN-γ, IL-2, IL-5, IL-17, IL-10, and IL-4) and the proliferation of splenic and CLN CD4+ T cells from mice infected with *S. pneumoniae* [122]. Moreover, the multi-epitope vaccine CAD, composed of the B- and T-cell epitopes of the pneumococcal surface protein C (PspC), PsaA, and PhtD proteins, was designed and constructed, and its immunogenicity and protection effects were assessed in mice [54,123]. The data suggested that the CAD vaccine elicited high IgG antibody titers and high levels of production of IFN-γ, IL-4, and IL-17 cytokines in serum; provided good protection effects; and decreased bacterial loads in blood and spleen in mice intraperitoneally challenged with *S. pneumoniae*. The CAD vaccine also demonstrated complement-mediated pneumococcus clearance ability [54].

Another epitope vaccine, PiuA-PlyD4, composed of B- and Th-cell epitopes derived from lipoprotein PiuA with PlyD4 (C-terminal D4 domain (AA_360–471_) of Ply) as an adjuvant, produced higher IgG antibody titers and cytokine production levels (Th1, Th2, and Th17) in immunized mice compared to control groups [59]. This vaccine also showed a higher clearance rate of pneumococci in the nasal cavity and lungs in a pneumonia infection model and increased survival rates in an intraperitoneal challenge sepsis model. Additionally, the PiuA-PlyD4 fusion protein antiserum inhibited the adherence of bacteria to the A549 cell line [59].

Additionally, ten immunodominant B-cell epitopes derived from six pneumococcal virulence proteins (CbpD, PhtD, PhtE, PspA, plasminogen and fibronectin-binding protein B (PfbB), and zinc metalloproteinase B (ZmpB)) were identified, which were highly conserved among different pneumococcal serotypes [124]. However, only three peptides (CbpD-pep4, PhtD-pep19, and PhtE-pep40) were broadly recognized by invasive pneumococcal disease (IPD) patient sera. Purified antibodies against four peptides (CbpD-pep4, PhtD-pep19, PhtE-pep40, and ZmpB-pep125) were effective in killing pneumococci in phagocytic cells [69,124]. Mice immunized with these peptides alone or in combination induced higher IgG antibody titers and exhibited longer survival times than control groups in an intraperitoneal challenge sepsis model. Notably, PhD-pep19 and PhtE-pep40 exhibited superior immunogenic and protective efficacy compared to CbpD-pep4 and ZmpB-pep125 [69]. These studies collectively highlight the potential of epitope-based vaccines as effective alternatives to traditional pneumococcal vaccines, offering broader serotype coverage and enhanced immunogenicity.

Several epitope-based vaccines mapped on single or multiple pneumococcal virulence proteins have been predicted and evaluated using bioinformatics tools. Examples include multi-epitope vaccines comprising B- and T-cell epitopes mapped on the Ply, PsaA, PspA, and PspK proteins [125] or derived from PspC [126]. Additionally, a new report details a multi-epitope vaccine composed of T-cell epitopes targeting the PspA, PspC, and CbpA of *S. pneumoniae* and the outer membrane proteins OmpA and OmpW of *K. pneumoniae*, which effectively combated pneumococcal infection [127]. Nevertheless, *in vitro* and *in vivo* experimental validation is necessary to confirm the potency of these epitope-based vaccines against multiple pneumococcal strains.

### 3.3. Advances in the Development of Epitope-Based Vaccines Against S. pyogenes

*S. pyogenes* (also known as Group A streptococci, GAS) ranks among the top ten causes of human mortality from infectious diseases. It is responsible for a wide range of illnesses, including pharyngitis, scarlet fever, impetigo, sepsis, necrotizing fasciitis, streptococcal toxic shock syndrome, acute rheumatic fever (ARF), and rheumatic heart disease (RHD) [128,129]. These infections and their post-infection complications lead to more than 500,000 GAS-associated deaths annually [128,129]. To date, no vaccine has been approved to prevent GAS infections, although several candidates are under development. An ideal GAS vaccine should avoid inducing autoimmune pathology and provide broad protection across most GAS serotypes [130,131].

The M protein is a major virulence factor and protective antigen of *S. pyogenes*, characterized by a highly polymorphic N-terminal region and a conserved C-terminal region across different serotypes [132]. Due to the potential of the M protein to induce autoimmune responses, current research has shifted towards developing multi-epitope vaccines that exclude the autoimmune regions of the M protein. For instance, the StreptInCor vaccine incorporates both T- and B-cell epitopes mapped to the natural C-terminal portion of the M5 protein, inducing high IgG titers, splenocyte proliferation, and protective effects against *S. pyogenes* infection in a mouse intraperitoneal challenge sepsis model [82,133]. Its antiserum has also demonstrated the ability to inhibit bacterial adhesion and invasion into HEp-2 cells. Similarly, the multi-epitope vaccine F7M5, composed of seven predominant epitopes of fibronectin-binding protein (FbaA) and five non-tissue cross-reactive epitopes of M protein from four predominant serotypes associated with ARF in China, elicited high IgG titers and a balanced IgG1/IgG2a response. This vaccine provides strong protective immunity without cross-reactivity in mouse intraperitoneal challenge sepsis models [83].

To enhance coverage across more GAS subtypes, several M protein-based multi-epitope vaccines have been developed. A well-known example is the J8-DT vaccine, which provides profound protection against multiple GAS strains in mouse skin infection and bacteremia models; however, it has shown ineffectiveness against highly virulent CovR/S mutant strains [134]. Meanwhile, combining J8-DT with *S. pyogenes* cell envelope proteinase (SpyCEP) or a minimal epitope (S2) of SpyCEP has been shown to induce strong protection against CovR/S mutant strains in mouse skin infection models [65,134].

Currently, 9-valent and 12-valent epitope-based vaccines targeting conserved linear B-cell epitopes from the N-terminal regions of M proteins have been designed to cover 117 epidemiologically relevant GAS subtypes, inducing strong IgG responses in rabbits [135]. The antisera from these rabbits exhibit opsonophagocytic-mediated bactericidal activity against a subset of vaccine and non-vaccine M types of *S. pyogenes* [135]. Furthermore, to enhance immune responses, these multi-epitope vaccines require the incorporation of adjuvants (immune stimulants) and/or effective delivery systems. Azuar et al. reported that several multi-epitope vaccines containing B-cell epitopes (J8, PL1, and 88/30) derived from the M protein, when delivered *via* poly(hydrophobic amino acid) conjugates, trigger high IgG titers [136]. Despite J8-DT and StreptInCor entering phase I clinical trials in 2013 and 2016, respectively [137,138], the challenge of the multi-epitope vaccines based on the M protein remains in their limited GAS subtype coverage.

Given the limitations of M protein-based vaccines, several multi-epitope vaccines based on non-M protein antigens have been explored. The multi-epitope vaccine (FSBM) contains four different epitopes mapped on the fibronectin-binding repeats domain of streptococcal fibronectin-binding protein (Sfb1), the C-terminal immunogenic segment of streptolysin S (SLS), the C3-binding motif of streptococcal pyrogenic exotoxin B (SpeB), and the C-terminal conserved segment of the M protein [66]. It induces IgG antibodies that opsonize various *S. pyogenes* serotypes, reduces internalization into A549 or HMEC-1 cells, and neutralizes the hemolytic activity of SLS [66]. Furthermore, vaccination with the rFSBM protein reduces skin lesions and improves survival rates in mouse models of skin infection and intraperitoneal challenge sepsis [66].

In addition, various multi-epitope vaccines based on non-M proteins have been predicted and designed using bioinformatics tools: for instance, a multi-epitope vaccine containing seven T-cell epitopes from three surface proteins (SpyCEP, Sfb1, and protein F) [139] and MEBSV, a multi-epitope vaccine composed of 5LBL, 9CTL, and 4HTL epitopes derived from four highly proteins (ATP synthase subunit a (AtpB), phospho-N-acetylmuramoyl-pentapeptide-transferase (MraY), glycerol-3-phosphate acyltransferase (PlsY), and transport permease protein (RgpCc)) [140]. Despite promising results, further experimental validation is necessary to confirm the immunogenicity and protective efficacy of these multi-epitope vaccine constructs against a broad range of GAS subtypes.

### 3.4. Advances in the Development of Epitope-Based Vaccines Against K. pneumoniae

*K. pneumoniae* is a major cause of nosocomial infections worldwide, responsible for various infections such as UTIs, cystitis, pneumonia, surgical wound infections, endocarditis, and septicemia [95,141]. The rising incidence of multidrug-resistant (MDR) *K. pneumoniae* has made it a significant clinical and public health challenge. Despite the high morbidity and mortality associated with *K. pneumoniae* infection, especially in immunocompromised individuals, there is currently no effective vaccine available to counter this pathogen.

Outer membrane proteins (OMPs) such as OmpA, OmpK36, OmpK17, and OmpW are attractive candidate antigens for *K. pneumoniae* due to their immunogenicity and protective efficacy, which have been confirmed in animal models [142,143,144]. A multi-epitope vaccine named r-AK36, mapped on the epitopes of the OmpA and OmpK36 proteins, was reported in 2017 [84]. It induced high levels of IgG, IgM, IL-2, and IFN-γ, as well as lymphocyte proliferation, resulting in significant effectiveness in preventing *K. pneumoniae* infection in a mouse sepsis model [84]. Moreover, anti-r-AK36 antibodies demonstrated antimicrobial effects and biofilm inhibition against *K. pneumoniae*. Utilizing bioinformatics tools, Liao et al. designed a multi-epitope vaccine named mHla-EpiVac, based on ferric enterobactin protein (FepA), OmpA, and OmpW and with the alpha-hemolysin mutant (mHla) as a heptamer platform [85]. They evaluated its immunogenicity and protective effects in a mouse acute pneumonia model, concluding that mHla-EpiVac could activate and mature BMDCs, eliciting robust humoral and cellular immune responses and providing protective efficacy against *K. pneumoniae* lung infection.

Fimbria adhesin protein MrkD has also been used as a target antigen for designing epitope-based vaccines. Li et al. introduced three Th-cell epitopes (M_221–235_, M_175–189_, M_264–278_) of MrkD [145]. The M_221–235_ and M_175–189_ epitope peptides induced high IL-4 levels, while the M_264–278_ epitope peptide increased IFN-γ levels in splenic lymphocytes. All three epitopes evoked a CD4+ T-cell response in immunized mice. However, it remains to be evaluated whether these epitope peptides can provide protective efficacy against *K. pneumoniae* infection.

Likewise, various epitope-based vaccines for *K. pneumoniae* have been predicted and designed, though they lack *in vivo* validation. Examples include multi-epitope vaccines based on B-cell epitopes from proteins such as the copper silver efflux system outer membrane protein (CusC), OmpN, Fe+ enterobactin transporter substrate binding protein (FepB), zinc transporter substrate binding protein (ZnuA), ribonuclease HI (RnhA), tellurite-resistant methyltransferase (TehB), and hypothetical proteins (WP_002918223 and WP_002892366) [146], as well as fimbriae proteins (FimA, FimF, FimG, FimH) [147]; the multi-epitope vaccine based on T-cell epitopes from FimA, FimF, FimG, and FimH [148]; and the multi-epitope vaccines including B- and T-cell epitopes from OmpA, CusC, phosphoporin PhoE, peptidoglycan-associated lipoprotein (Pal) [149], type 3 fimbrial protein MrkA [150], FepA [151], capsular polysaccharide (CPS) protein [152], putative TonB-dependent siderophore receptor (KPHS_13560), β-1,4-mannanase (KPHS_41440), MrkD, putative cellulose synthase and BcsC protein (KPHS_50480) [153], PhoE, Pal, FepA, OmpW, Fiu, SlyB, Lpp, OmpN, NlpD, KdgM, DamX, YiaD [154], OmpK17 [155], MrkD, iron-regulated lipid membrane polypeptides (IutA), RmpA [156], OmpA, OmpC, OmpX, OmpW, and OmpK37 [157].

Intriguingly, several multi-epitope vaccines were designed to combat co-infections involving *K. pneumoniae* and other bacteria: for example, a multi-epitope vaccine composed of the T-cell epitopes targeting nine antigens to fight *E. coli* (ExPEC), *Proteus mirabilis*, and *K. pneumonia* causing UTIs [158]. Other multi-epitope vaccines targeting the OmpA of *K. pneumonia* and Rv1698, the Rv1973 of *Mycobacterium tuberculosis* [159], FepA and OmpK35 for *K. pneumonia* and HasR, or OprF for *P. aeruginosa* [160] or mapped on OmpA, the OmpW of *K. pneumoniae*, or the PspA, PspC, and CbpA of *S. pneumoniae* [127] have also been reported. Although these multi-epitope vaccines show potential, their effectiveness in generating immune responses *in vivo* remains to be validated.

### 3.5. Advances in the Development of Epitope-Based Vaccines Against A. baumannii

*A. baumannii* is an emerging MDR pathogen that causes a variety of nosocomial infections, including pneumonia, meningitis, sepsis, endocarditis, skin infections, and urinary tract infections, particularly in intensive care unit (ICU) patients [161,162,163]. *A. baumannii* infection leads to significant mortality, especially in intensive care unit (ICU) patients. The significant mortality associated with *A. baumannii* infections and increasing antibiotic resistance underscore the urgent need for an effective vaccine. However, no vaccines against this bacterium have been marketed to date.

OMPs are integral membrane proteins that play crucial roles in biofilm formation, eukaryotic cell infection, antibiotic resistance, and the pathogenicity of *A. baumannii* [164,165]. Consequently, OMPs, such as OmpA, OmpC, OmpK, OmpW, OmpW2, Omp22, Omp33–36, BamA, LptD, CarO, OprD, and PcTPRs1, have been proposed as suitable vaccine candidates capable of eliciting strong immune responses [166,167,168]. Mehdinejadiani’s team predicted the T- and B-cell epitopes of OmpA, synthesizing five epitope peptides (P1, P2, P3, P4, and P5) [63,169]. Peptide P1_24–50_ stimulated high IgG antibody titers and IFN-γ levels, improving the survival rate of mice in sepsis and pneumonia models after both passive and active immunization. The other multi-epitope polypeptide rOmp22 comprises three optimal B-cell epitopes and two optimal T-cell epitopes from the Omp22 protein and is encapsulated in chitosan (CS) and poly (lactic-co-glycolic) acid (PLGA) nanoparticles (NPs) [62]. BALB/c mice immunized with CS-PLGA-rOmp22 presented high levels of rOmp22-specific IgG in the serum and IFN-γ in the splenocyte supernatant, which reduced lung injury and bacterial burdens in the lungs and blood and improved survival rates in a pneumonia model. Another epitope vaccine containing B- and T-cell epitopes (amino acids 290–390) from the PcTPRs1 protein decreased the mortality rate of the immunized mice infected with *A. baumannii* in a pneumonia model [86]. And a single 15-amino-acid epitope peptide, containing both linear B- and T-cell epitopes of OmpW, induced cell proliferation but did not stimulate IFN-γ production in patient-derived peripheral blood mononuclear cells [170]. In addition, an epitope vaccine that contains epitope 1_55–90_ and epitope 3_276–309_ of OmpW2 induced high IgG titers, decreased the mortality rate, and reduced the bacterial burden in the lungs, liver, kidneys, and spleen in a mouse pneumonia model [87].

Moreover, the first reported multi-epitope assembly peptide (MEP) vaccine against *A. baumannii* was based on the Ata protein and B- and T-cell epitopes of the outer membrane proteins FilF and NucAb [88]. This vaccine induced high levels of IgG antibodies and provided protective immune responses in a mouse sepsis model. Another recombinant antigen epitope vaccine (RAE) composed of three epitopes (B-cell epitope 2, CD8+ T-cell epitope 7, and CD4+ T-cell epitope 11) based on the efflux pump MacB protein stimulated a Th1 immune response, with high IgG antibody titers and significant IFN-γ and IL-2 levels, and provided partial immune protection in a mouse sepsis model [89]. Raoufi et al. reported a multiple-epitope vaccine containing three epitopes from the DcaP porin that elicited high IgG antibody titers and partial protection in a mouse pneumonia model [90].

Notably, several multi-epitope vaccines against *A. baumannii* have been predicted using bioinformatics tools, but they lack *in vitro* and *in vivo* experimental validation. These include vaccines based on the following: B-cell epitopes from Baumannii acinetobactin utilization (BauA) [171]; OmpA [172]; OmpK [173]; T-cell epitopes from OmpK [174]; B- and T-cell epitopes from outer membrane nuclease NucAb [175]; Tol-Pal system protein TolB [176]; polysaccharide export outer membrane protein (EpsA) and chaperone–usher pathway protein B (CsuB) [177]; TonB-dependent copper receptor (EEX02051.1) [178]; outer membrane protein assembly factor (BamA), fimbrial biogenesis outer membrane usher protein (FimD), and type IV secretion protein (Rhs) [179]; two-component system (TCS) histidine kinase GacS [180]; extracellular and outer membrane proteins Hcp and OmpC [181]; TonB, OmpA, and OprD [182]; pilus assembly protein FilF [183]; TCS-associated proteins BfmR and BfmS [184]; LPS assembly proteins LptE and LptD [185]; and TCS response regulator BaeR [186]. Despite promising results from various studies, challenges remain in developing effective epitope-based vaccines against *A. baumannii*. Further studies are needed to optimize and validate these vaccines in animal models and clinical trials to ensure they induce protective immune responses.

### 3.6. Advances in the Development of Epitope-Based Vaccines Against P. aeruginosa

*P. aeruginosa* is one of the most dangerous human pathogens globally, causing severe infections such as ventilator-associated pneumonia, skin infections, urinary tract infections, and sepsis [187,188]. It significantly contributes to mortality in hospitalized patients worldwide. Despite the alarming increase in multidrug resistance, no approved vaccines are available to prevent *P. aeruginosa* infections.

Recently, many epitope-based vaccines were reported for preventing the infection against *P. aeruginosa*. Gilleland’s lab synthesized several linear B-cell epitopes of outer membrane protein F (OprF) and evaluated their protective immune efficacy *in vivo* [91,189,190]. Notably, peptides 9 (or Epi6, TDAYNQKLSERRAN) and 10 (or Epi8, NATAEGRAINRRVE) elicited protective immune responses against *P. aeruginosa* in mice, highlighting their potential as vaccine candidates [91,189,190,191,192,193]. With regard to PilA, PilY1 and PilQ associated with type IV pili were selected as vaccine candidates. Gao et al. screened the epitope Ep_167–193_ from PilY1, delivered by macrophage membrane-coated PLGA nanoparticles, which induced a Th2 immune response and protective immunity against *P. aeruginosa* in a mouse pneumonia model [92].

More information on multi-epitope vaccines is available for *P. aeruginosa*. In 2020, Wang et al. developed the multi-epitope vaccine PVAC, comprising Th17-stimulating epitopes of the type 3 secretion system component PcrV and β-lactamase AmpC [93]. This vaccine elicited a humoral immune response and Th17 response, providing broad protective immunity against *P. aeruginosa* in a mouse pneumonia model. The Asadi Karam team constructed a multi-epitope vaccine containing B- and T-cell epitopes derived from PcrV and outer membrane protein E (OmpE) [94]. This vaccine triggered high IgG titers, decreased the load of *P. aeruginosa*, and induced protective immune responses in a rabbit urinary tract infection model. Another efficient multi-epitope vaccine derived from six protein surface components (antibiotic efflux pumps PA2837 and PA3521, chaperone–usher pathway components CupC2 and CupB3, penicillin-binding protein PBP1a/mrcA, and type 3 secretion system component PscC) was also developed by Asadi Karam’s team [67]. Encapsulated with silk fibroin nanoparticles, this vaccine induced high levels of IgG and IgA in serum and urine, as well as high cytokine levels of IFN-γ, IL-4, and IL-17, conferring both preventive and therapeutic effects in a mouse urinary tract infection model [67].

Likewise, several multi-epitope vaccines against *P. aeruginosa* have been predicted using bioinformatics tools, which evaluated their physicochemical features, antigenicity, toxicity, and allergenicity. These include the following: multi-epitope vaccines containing B- and T-cell epitopes from fusion proteins (PilQ_380–706_-PilA) [194], fructose bisphosphate aldolase (FBA) [195], amyloid-forming protein FapC [196], outer membrane proteins OprF and OprI [197], five proteins (polymerized flagellin subunits FliC, type-specific cap protein FliD, OprI, OprF, and PilA) [198], or OprF and heme acquisition protein HasR [160]. Despite promising predictions, the *in vivo* validation and clinical trials for these multi-epitope vaccines against *P. aeruginosa* are yet to be undertaken.

## 4. Current Challenges of Bacterial Epitope-Based Vaccines

Within the past decade, the design and verification of bacterial epitope-based vaccines have increased rapidly. These vaccines offer several advantages over traditional inactivated, live attenuated, protein, and DNA/RNA vaccines, such as reduced adverse effects, enhanced immune response efficiency, and optimized protective efficacy in a time- and cost-effective manner. As outlined in this review, several epitope-based vaccines have demonstrated good immunogenicity and protective effects against bacterial infections in animal models, with some even advancing to clinical trials. However, epitope-based vaccines remain in their infancy. As the next generation of vaccines for combating bacterial infection, epitope-based vaccines still face several significant challenges that must be addressed (Table 3).

First, although many programs for epitope prediction are available, determining the most effective tool remains unclear. For example, tools such as BCPred, BepiPred, ABCpred, and IEDB can be used to predict B-cell epitopes (BCEs). However, these tools often provide varying results for the same antigen, making it difficult to identify the most accurate prediction. A similar issue arises with T-cell epitope (TCE) predictions. To establish the best prediction tool, it is essential to perform independent tests using a standardized set of protocols across different programs, comparing predictions against experimentally validated results.

Second, the inaccuracies of epitope prediction tools pose another challenge in the development of effective epitope-based vaccines. The limited availability of data on B- and T-cell epitopes and the variability in immune responses often result in predicted vaccines that fail to induce protective effects in animal models. Artificial intelligence (AI), particularly ML, offers a promising solution. AI has been widely applied in bacterial infection diagnosis, antibacterial drug design, and vaccine development [199,200,201]. By leveraging AI, an ML model with a sufficiently large training dataset of “positive” epitopes (those that induce protective effects in animal models) and “negative” epitopes (those that do not) could significantly increase the accuracy of epitope prediction tools, reducing false-positive results.

Third, the lack of standardized protocols for assembling epitopes and selecting the appropriate epitopes, adjuvants, and linkers represents another hurdle. The construction of optimal epitope-based vaccines requires the careful selection of all three components. Different epitopes may need different adjuvants and linkers to achieve the best protective effects, and any misstep in their selection could negate the vaccine’s immunogenic potential. Vaccine formulations, dosages, and delivery systems also play critical roles. Currently, prediction tools for epitopes, adjuvants, and linkers operate independently, which leads to operational complexity and inconsistency in results. An integrated, AI-driven platform that uses a standardized set of design protocols could streamline the process, ensuring the better assembly of epitopes, adjuvants, linkers, and other vaccine components.

Fourth, most predicted epitope-based vaccines are validated only through *in silico* studies, with a minimal verification of their biosafety and true protective efficacy in animal models or clinical trials. Owing to time, financial, or legal constraints, few predicted vaccines undergo extensive testing. Recent advances in organoid technology, which allows for the creation of three-dimensional (3D) models replicating human organs such as the lungs, brain, heart, and kidneys, offer a promising *in vitro* alternative [202,203,204,205,206]. When coupled with AI to create “Organoid Intelligence” (OI), organoids can reduce the need for animal testing by providing a preclinical model for the experimental validation of vaccines [207]. Additionally, AI can aid in selecting suitable vaccine candidates, streamlining clinical trial recruitment, and simplifying trial design, ultimately reducing the time and cost required to bring vaccines to the market [199,208]. While *in vivo* animal testing and clinical trials remain essential, AI and OI provide promising avenues for accelerating vaccine development.

Despite progress in epitope-based vaccine development, obtaining regulatory approval from agencies such as the FDA and EMA remains challenging. Current frameworks lack specific guidelines, leading to uncertainty in the evaluation process. Collaboration among regulators, researchers, and industry stakeholders is essential to establish tailored guidelines for efficient and consistent approval.

## 5. Future Prospects

To fully harness the potential of rational epitope-based vaccines, future research must focus on developing an open access, integrated platform that combines AI technologies for each stage of the vaccine design process. This platform would cover target antigen selection, epitope prediction and selection, adjuvant identification, linker design, and the subsequent steps of formulating vaccine dosages and delivery systems. Such an AI-driven approach could significantly enhance accuracy, reduce false-positive vaccine candidates, and identify conserved epitopes across various bacterial subtypes and drug-resistant strains, paving the way for broad-spectrum vaccines with cross-protective efficacy.

In addition, an increasing number of predicted epitope-based vaccines could be validated using advanced organoid models, followed by animal models and clinical trials, ultimately resulting in patented vaccines available for commercial use in preventing and treating multiple bacterial infections. Moreover, exploring multivalent epitope-based vaccines, which target multiple antigens to prevent co-infections caused by different bacteria—such as those responsible for pneumonia—offers an intriguing direction for future research.

Another promising avenue is the development of personalized epitope-based vaccines, made possible through the integration of AI or OI with single-cell omics and synthetic biology technologies. While the high production costs of personalized vaccines pose a challenge for broad population applicability, advances in high-throughput technologies and AI-driven automation may gradually reduce costs, making personalized vaccines more accessible in the future.

However, the large-scale production of epitope-based vaccines faces significant financial and logistical barriers. First, the high costs associated with advanced technologies, such as AI-driven platforms, organoid models, and single-cell omics, may limit accessibility for many research institutions and pharmaceutical companies. Second, the complexity of epitope-based vaccine design, including precise antigen selection, adjuvant optimization, and delivery system formulation, demands significant investment in infrastructure and expertise. Third, regulatory approval processes for novel vaccine platforms can be time-consuming and costly, further delaying commercialization. Lastly, the logistics of the manufacturing, storage, and distribution of epitope-based vaccines, particularly those requiring cold chain management, pose additional challenges that must be addressed to ensure global accessibility. By addressing current challenges and incorporating cutting-edge technologies, the creation of effective and reliable epitope-based vaccines can be accelerated, potentially revolutionizing the prevention and treatment of bacterial infections.

## Figures and Tables

**Table 1:** The advantages of epitope-based vaccines over traditional vaccines.

Aspect	Traditional Vaccines	Epitope-Based Vaccines
Ingredient	Composed of complex whole pathogens or large fragments	Simple and well defined, consisting of specific epitopes
Target Specificity	Broad, less specific	Highly specific
Safety	Higher risk of adverse reactions	Minimal adverse reactions
Production Process	Complex, time-consuming, and costly	Streamlined and cost-effective
Adaptability	Limited flexibility, especially for rapid updates	Easily adaptable to new strains or variants

**Table 2:** Summary of target antigens and their localization, epitope types, and *in vivo* validated animal models of epitope-based vaccines with known protective effects against *S. aureus*, *S. pneumoniae*, *S. pyogenes*, *K. pneumoniae*, *A. baumannii*, and *P. aeruginosa*.

Bacteria	Target Antigens *	Localization of Target Antigens	Names of Epitope-Based Vaccines and Epitope Types	*In Vivo* Validated Animal Models	Selected References
*S. aureus*	SEB	Surface protein	B-cell epitopes	Mouse bacteremia model	[70,71]
	MntC	Cell membrane	B-cell epitopes	Mouse bacteremia model	[71,72]
	Hla	Surface protein	B-cell epitopes	Mouse bacteremia model	[71,73]
	IsdB	Surface protein	B-cell epitopes	Mouse bacteremia model	[71,73]
	GapC	Surface protein	B-cell epitopes	Mouse sepsis model	[74,75]
	FnBPA	Cell wall	B-cell epitopes	Mouse sepsis model	[76]
	TRAP	Cell wall	Th-cell epitopes	Mouse sepsis model	[77]
	PGN	Cell wall	T-cell epitopes	Mouse bacteremia model	[78]
	Plc	Secreted protein	B- and T-cell epitopes	Mouse sepsis model	[79]
	CgoX	Cytoplasm	B-cell epitopes	Mouse sepsis model	[80]
*S. pneumoniae*	PspA	Surface protein	B-cell epitopes	Mouse bacteremia model	[64]
	PspA	Surface protein	B- and Th-cell epitopes	Mouse sepsis model	[81]
	PhtD	Surface protein			
	PspC	Surface protein	CAD vaccine: B- and T-cell epitopes	Mouse sepsis model	[54]
	PsaA	Surface protein			
	PhtD	Surface protein			
	PiuA	Surface protein	PiuA-PlyD4: B- and Th-cell epitopes	Mouse pneumonia model and sepsis model	[59]
	CbpD	Surface protein	B-cell epitopes	Mouse sepsis model	[69]
	PhtD	Surface protein			
	PhtE	Surface protein			
	PspA	Surface protein			
*S. pyogenes*	M protein	Cell wall	StreptInCor: B- and T-cell epitopes	Mouse sepsis model	[82]
	FbaA	Surface protein	F7M5: uncertain epitope types	Mouse sepsis model	[83]
	M protein	Cell wall			
	M protein	Cell wall	J8-DT/S2: B-cell epitopes	Mouse skin infection model	[65]
	SpyCEP	Cell wall			
	Sfb1	Cell wall	FSBM: uncertain epitope types	Mouse skin infection and sepsis model	[66]
	SLS	Surface protein			
	SpeB	Secreted protein			
	M protein	Cell wall			
*K. pneumoniae*	OmpA	Cell outer membrane	r-AK36: uncertain epitope types	Mouse sepsis model	[84]
	OmpK36	Cell outer membrane			
	FepA	Cell outer membrane	mHla-EpiVac: B- and T-cell epitopes	Mouse acute pneumonia model	[85]
	OmpA	Cell outer membrane			
	OmpW	Cell outer membrane			
*A. baumannii*	OmpA	Cell outer membrane	B- and T-cell epitopes	Mouse sepsis model and pneumonia model	[63]
	rOmp22	Cell outer membrane	B- and T-cell epitopes	Mouse pneumonia model	[62]
	PcTPRs1	Cell outer membrane	B- and T-cell epitopes	Mouse pneumonia model	[86]
	OmpW2	Cell outer membrane	B- and T-cell epitopes	Mouse pneumonia model	[87]
	FilF	Cell outer membrane	B- and T-cell epitopes	Mouse sepsis model	[88]
	NucAb	Cell outer membrane			
	MacB	Cell membrane	RAE: B- and T-cell epitopes	Mouse sepsis model	[89]
	DcaP	Cell outer membrane	B- and T-cell epitopes	Mice pneumonia model	[90]
*P. aeruginosa*	OprF	Cell outer membrane	B-cell epitopes	Mouse pneumonia model	[91]
	PilY1	Cytoplasm/Cell membrane	Ep_167–193_: uncertain epitope types	Mouse pneumonia model	[92]
	PcrV	Secreted protein	Th17-cell epitopes	Mouse pneumonia model	[93]
	AmpC	Periplasm			
	PcrV	Secreted protein	B- and T-cell epitopes	Rabbit UTI model	[94]
	OmpE	Cell outer membrane			
	PA2837	Cell outer membrane	B- and T-cell epitopes	Mouse UTI model	[67]
	PA3521	Cell outer membrane			
	CupC2	Periplasm			
	CupB3	Cell outer membrane			
	PBP1a	Cell membrane			
	PscC	Cell outer membrane			

* SEB, Staphylococcal enterotoxin B; MntC, manganese binding surface lipoprotein C; Hla, alpha-toxin; IsdB, iron surface determinant B; GapC, glyceraldehyde-3-phosphate dehydrogenase C; FnBPA, fibronectin-binding protein A; TRAP, RNAIII Activating Protein; PGN, peptidoglycan; Plc, phosphatidylinositol phosphodiesterase; CgoX, coproporphyrinogen III oxidase; PspA, pneumococcal surface protein A; PhtD, pneumococcal histidine triad protein D; PspC, pneumococcal surface protein C; PsaA, pneumococcal surface adhesin A; PiuA, lipoprotein of the iron uptake ABC transporter; CbpD, choline-binding protein D; PhtE, pneumococcal histidine triad protein E; FbaA, fibronectin-binding protein; SpyCEP, *S. pyogenes* cell envelope proteinase; Sfb1, streptococcal fibronectin-binding protein; SLS, streptolysin S; SpeB, streptococcal pyrogenic exotoxin B; OmpA, outer membrane protein A; OmpK36, outer membrane protein K36; FepA, ferric enterobactin protein; OmpW, outer membrane protein W; rOmp22, outer membrane protein 22; PcTPRs1, outer membrane protein; OmpW2, outer membrane protein W2; FilF, outer membrane protein; NucAb, outer membrane protein; MacB, efflux pump; DcaP, porin; OprF, outer membrane protein F; PilY1, type IV pilus biogenesis factor; PcrV, type III secretion protein; AmpC, β-lactamase; OmpE, outer membrane protein E; PA2837, antibiotic efflux pump; PA3521, antibiotic efflux pump; CupC2, chaperone–usher pathway component; CupB3, chaperone–usher pathway component; PBP1a, penicillin-binding protein; PscC, type III secretion system secretin.

**Table 3:** Summary of challenges and potential strategies in bacterial epitope-based vaccine development.

	Challenge	Potential Strategy
1	Best tools for epitope prediction are unclear	Utilize multiple programs to predict epitopes for the same antigen, followed by experimental validation.
2	Inaccuracies in epitope prediction tools	Implement machine learning (ML) models with extensive “positive” and “negative” training datasets to improve prediction accuracy.
3	Lack of uniform standards for epitope assembly in vaccine construction	Develop an open access, integrated AI platform with standardized design protocols for assembling epitopes, adjuvants, and linkers.
4	Predicted vaccines often lack *in vivo* validation and clinical trials	Leverage AI and OI to reduce the time and cost of bringing vaccines to market.
5	Regulatory hurdles for epitope-based vaccine approval	Develop clear regulatory guidelines and enhance stakeholder collaboration to accelerate epitope-based vaccine approval.
